# Supplementary material for: Expanding the Toolbox for Genetic Manipulation in Pseudogymnoascus: RNAi-Mediated Silencing and CRISPR/Cas9-Mediated Disruption of a Polyketide Synthase Gene Involved in Red Pigment Production in P. verrucosus
Source: J Fungi (Basel). 2024 Feb 16;10(2):157. doi: 10.3390/jof10020157 (PMC10889956; doi:10.3390/jof10020157)
Supplement: Supplementary file 1 [file jof-10-00157-s001.zip › jof-2727775-supplementary.pdf]

Supplementary Material

Table S1. Currently accepted *Pseudogymnoascus* species and their MycoBank identifiers. Species are listed in alphabetical order. Data sourced from the MycoBank website (www.mycobank.org) on January 25, 2024.

N°	Species name	Mycobank identifier
1	*Pseudogymnoascus alpinus*	115510
2	*Pseudogymnoascus antarcticus*	838958
3	*Pseudogymnoascus appendiculatus*	501328
4	*Pseudogymnoascus australis*	838968
5	*Pseudogymnoascus botryoides*	844164
6	*Pseudogymnoascus campensis*	846366
7	*Pseudogymnoascus camphorae*	844165
8	*Pseudogymnoascus carnis*	804768
9	*Pseudogymnoascus catenatus*	840436
10	*Pseudogymnoascus caucasicus*	337766
11	*Pseudogymnoascus cavicola*	840116
12	*Pseudogymnoascus destructans*	804767
13	*Pseudogymnoascus fujianensis*	840437
14	*Pseudogymnoascus griseus*	838969
15	*Pseudogymnoascus guiyangensis*	901027
16	*Pseudogymnoascus guizhouensis*	835716
17	*Pseudogymnoascus hyalinus*	901023
18	*Pseudogymnoascus lanuginosus*	838970
19	*Pseudogymnoascus lindneri*	832750
20	*Pseudogymnoascus palmeri*	837413
21	*Pseudogymnoascus pannorum*	804769
22	*Pseudogymnoascus papyriferae*	844166
23	*Pseudogymnoascus rhousiogongylinus*	901028
24	*Pseudogymnoascus roseus*	276803
25	*Pseudogymnoascus shaanxiensis*	835715
26	*Pseudogymnoascus sinensis*	835717
27	*Pseudogymnoascus turneri*	832738
28	*Pseudogymnoascus verrucosus*	356754
29	*Pseudogymnoascus yunnanensis*	840438
30	*Pseudogymnoascus zhejiangensis*	840439
31	*Pseudogymnoascus zongqii*	844168

Table S2. Primers used in this work.

Name of the primer	Sequence (5´--- 3´)	Used for:	Reference
P1-KpnI<br><br>P2-pgdh-hph	GCGGATAACAATTTCACACAGGAAACAGCAGATTGCGACGGCGTATTGC<br><br>CTCGACAGACGTCGCGGTGAGTTCAGGCATGTCTGAAGGGGAGGATTGAT	Amplification of P*gdh* promoter from plasmid pJL43-RNAi	This work
P3-pgdh-hph<br><br>P4-HindIII	ATGCCTGAACTCACCGCGAC<br><br>GTAACGCCAGGGTTTTCCCAGTCACGACGAAGCTTAATGTGTGTCCTGTAGGCTT	Amplification of *hph* gene and T*trpC* terminator from plasmid pAN7-1	This work
azpA-RNAi-Fw<br><br>azpA-RNAi-Rv	AGACTATCTAGAGTACTTCCTTACGGGATACG<br><br>AGACTATCTAGAGAGACCTCAGTTGCTCTTTC	Amplification of RNAi target sequence of *azpA* gene.	This work
RNAi-conf-fw<br><br>RNAi-conf-rv	GCATGCCATTAACCTAGG<br><br>ACGGTGGCTGAAGATTC	Amplification of interference cassette (1619 - 1200pb)	This work
Seq-Cas9-azpA-fw<br>Seq-Cas9-azpA-rv	TATTTAGGCAGCCCACACTT<br>TATGACCCGTCTCAAACCAG	Amplification of the target region of *azpA*	This work
qRT-btub-Fw<br>qRT-btub-Rv	GAACTCCTCACGGATCTTGG<br>TCCAAGGTTTCCAGATCACC	b-tubulin gene expression analysis by qRT-PCR	[70]
azpA-Q-Fw<br>azpA-Q-Rv	AGACCATAGCACAGCCAACAAG<br>TTCCATCAGTCGCTCCAGTCAA	*azpA* gene expression analysis by qRT-PCR	This work

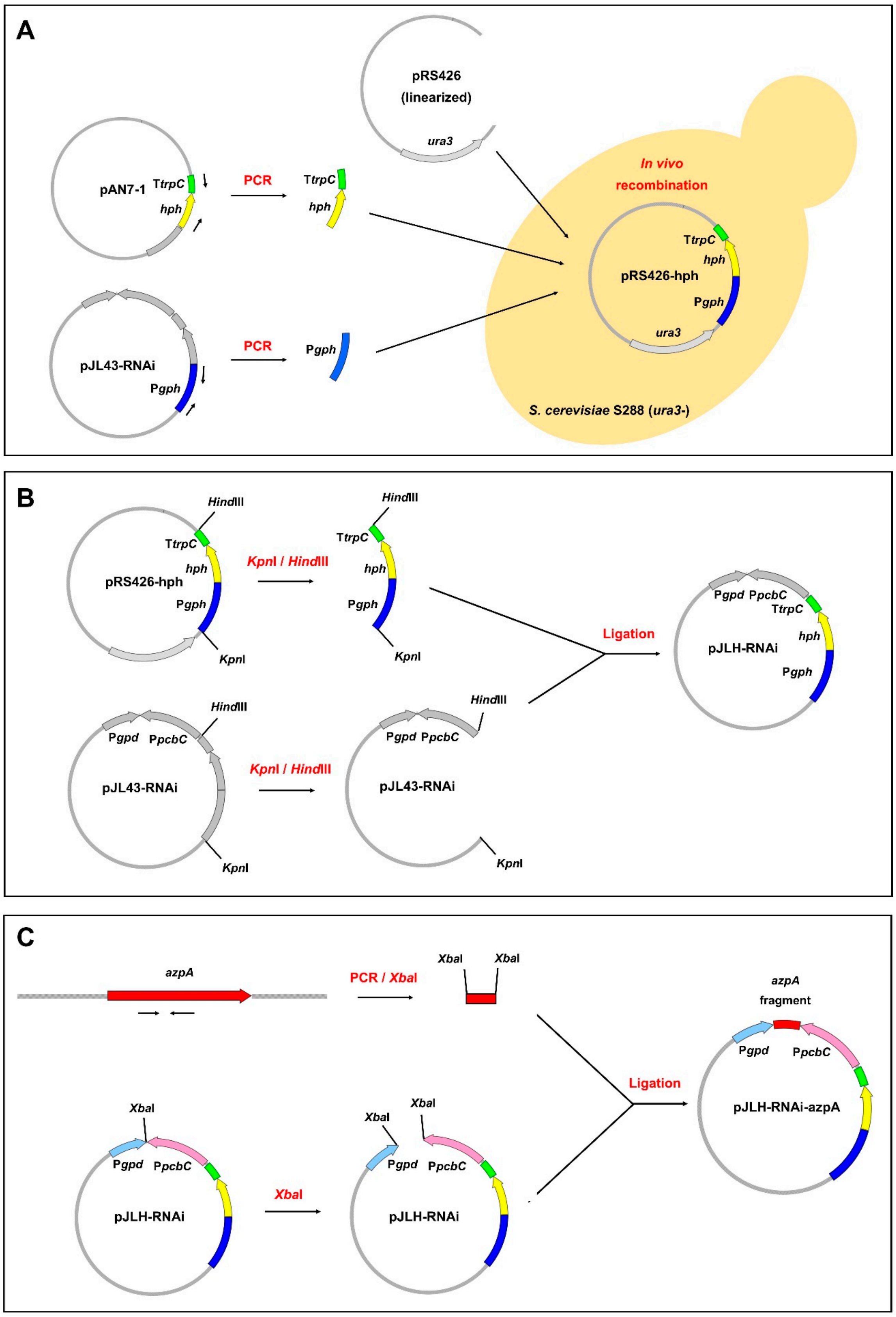

Figure S1. Schematic representation depicting the construction process of plasmid pJLH-RNAi-azpA for RNA-mediated silencing of *azpA* gene. (A). Assembly of the hygromycin resistance cassette. The P*gdh* promoter from *Aspergillus awamori* was obtained by PCR using pJL43-RNAi as the template. In parallel, the *hph* gene conferring hygromycin resistance, along with the TtrpC terminator from *Aspergillus nidulans*, was amplified from plasmid pAN7-1. Both PCR fragments were subjected to *in vivo* recombination in *Saccharomyces cerevisiae* using linearized plasmid pRS426, giving rise to pRS426-hph. **(B).** The hygromycin resistance cassette was released from pRS426-hph by *Kpn*I and *Hind*III digestion and subsequently ligated into plasmid pJL43-RNAi digested with the same enzymes, giving rise to pJLH-RNAi. **(C)** Finally, a 413 bp fragment from the *azpA* gene from *P. verrucosus* FAE27 was amplified by PCR from genomic DNA and digested with *Xba*I. The fragment was subsequently ligated into pJLH-RNAi, previously digested with the same enzyme, thus giving rise to the final plasmid pJLH-RNAi-azpA. For simplicity, the plasmid illustrations do not depict the regions required for *E. coli* transformation. Please note that the drawings are not to scale. For further details, see the “Materials and Methods” section in the main text.

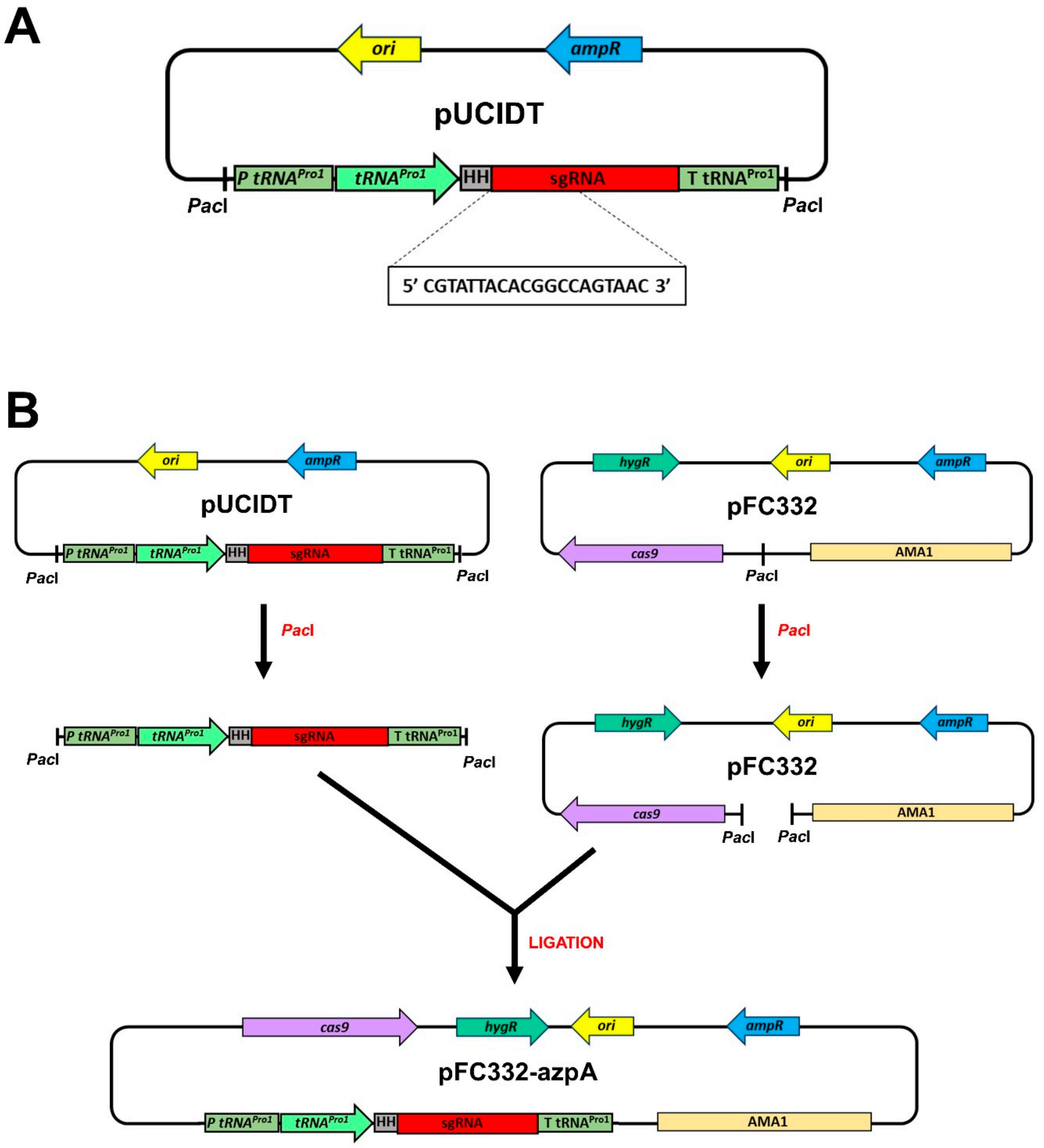

Figure S2. (A). Schematic representation of the sgRNA expression cassette for CRISPR-Cas9 disruption. The cassette was synthesized and cloned in pUCIDT by Integrated DNA Technologies. The cassette encompasses the promoter (P $tRNA^{Pro1}$) and gene sequence ($tRNA^{Pro1}$) of the proline-tRNA from *A. niger*, a hammerhead ribozyme sequence (HH), the sgRNA (which includes the target sequence 5′CGTATTACACGGCCAGTAAC 3′), and the terminator of the proline-tRNA (T $tRNA^{Pro1}$). The cassette also includes *Pac*I restriction sites at both ends for subsequent cloning. The depiction of pUCIDT includes the ampicillin resistance gene (ampR) and the replication origin of *E. coli* (ori). **(B). Construction of plasmid pFC332-azpA for *azpA* disruption by CRISPR-Cas9.** The sgRNA expression cassette was released from pUCIDT by *Pac*I digestion and subsequently ligated into plasmid pFC332 digested with the same enzyme, giving rise to final plasmid pFC332-azpA. As appropriate, the schemes include the ampicillin resistance gene (ampR) and the replication origin of *E. coli* (ori), the *cas9* gene, the AMA1 region for autonomous replication in fungi, and the hygromycin resistance gene (hygR). Please note that the drawings are not to scale. For details, see the "Materials and Methods" section in the main text.

A

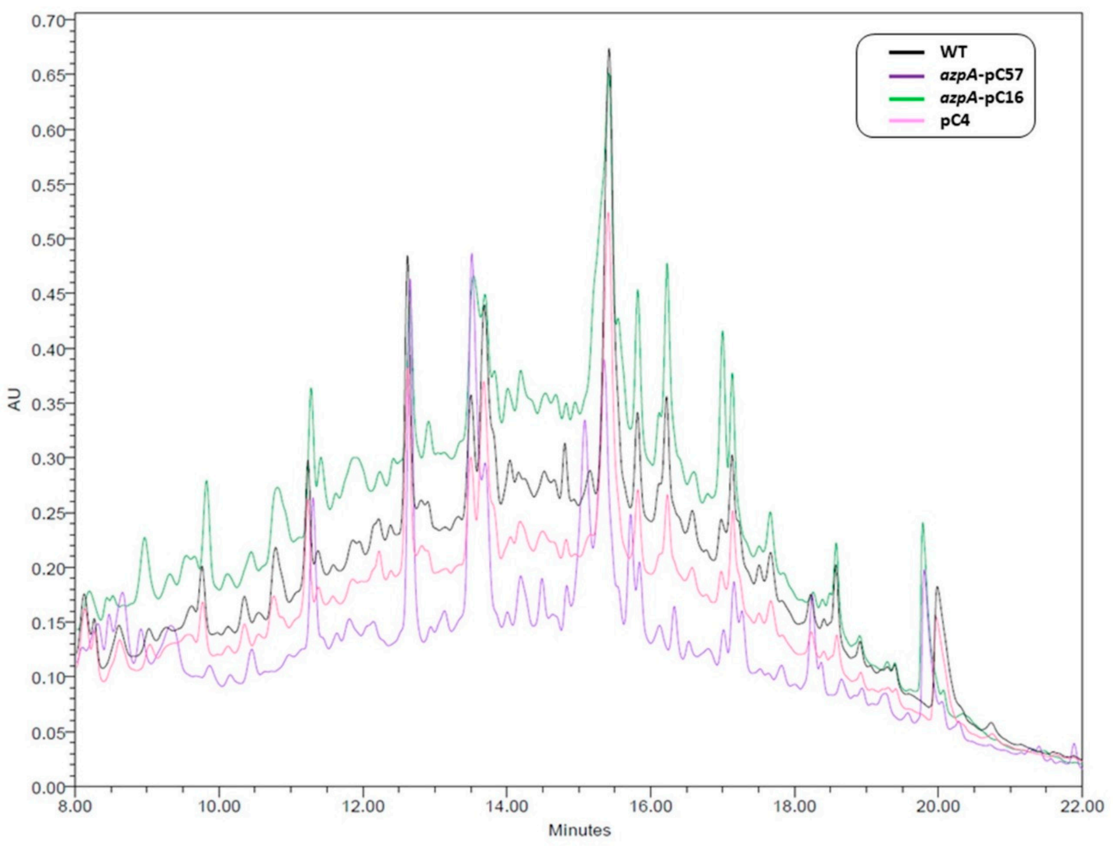

B

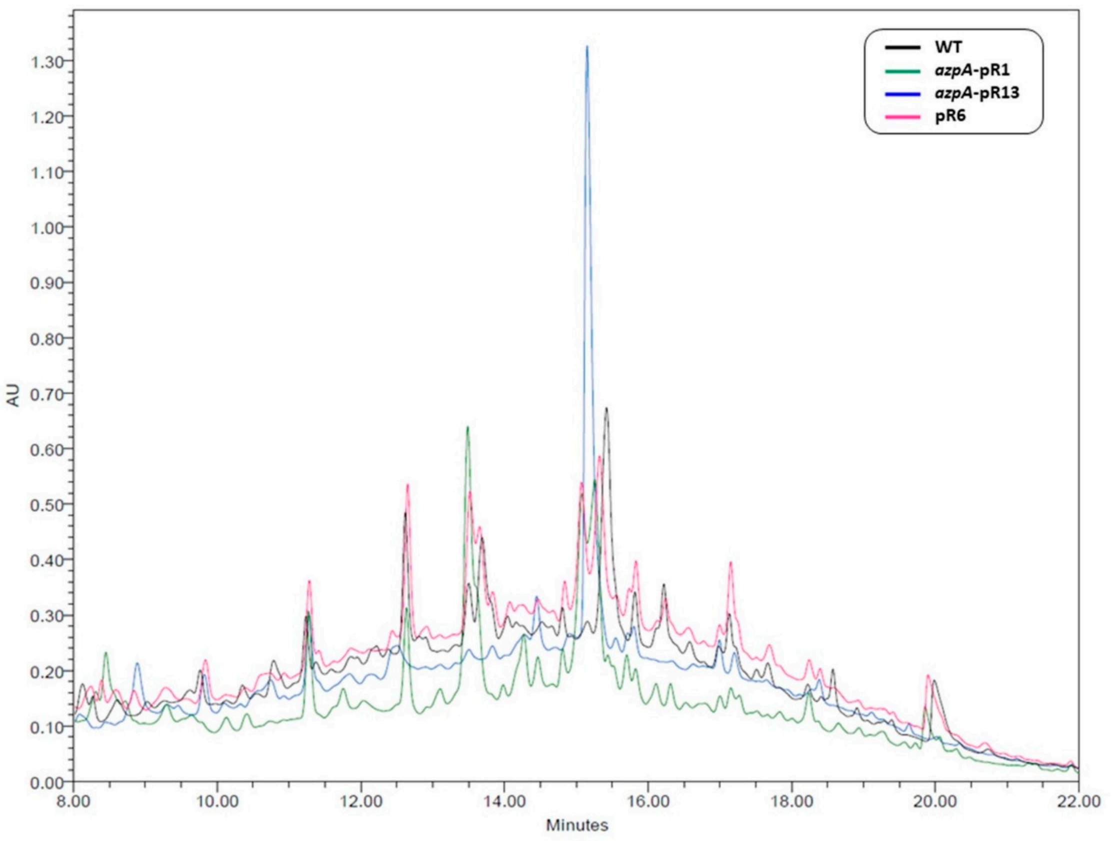

Figure S3. Comparative analysis of specialized metabolite profiles of *P. verrucosus* FAE27 azpA transformants at 254 nm. (A). Metabolic profile at 254 nm of *P. verrucosus* FAE27 (WT), and transformants obtained by CRISPR-Cas methodology. All the red transformants got using pFC332-azpA showed the same metabolic profile. As representative of them, was choice strain azpA-pC16. Likewise, all red transformants got using pFC332 showed the same profile. As representative of them, was choice strain pC4. Finally, all the white transformants also presented the same profile. As representative of them, was choice strain azpA-pC57. **(B).** Metabolic profile at 254 nm of *P. verrucosus* FAE27 (WT), and azpA transformants obtained by RNAi methodology. All the red transformants got using pJLH-RNAi-azpA showed the same metabolic profile. As representative of them, was choice strain azpA-pR1. Likewise, all red transformants got using pJLH-RNAi showed the same profile. As representative of them, was choice strain pR6. Finally, all the white transformants also presented the same profile. As representative of them, was choice strain azpA-pR1.
